# Enhanced Islet Cell Nucleomegaly Defines Diffuse Congenital Hyperinsulinism in Infancy but Not Other Forms of the Disease

**DOI:** 10.1093/ajcp/aqw075

**Published:** 2016-06-22

**Authors:** Bing Han, Melanie Newbould, Gauri Batra, Edmund Cheesman, Ross J. Craigie, Zainab Mohamed, Lindsey Rigby, Raja Padidela, Mars Skae, Aleksandr Mironov, Tobias Starborg, Karl E. Kadler, Karen E. Cosgrove, Indraneel Banerjee, Mark J. Dunne

**Affiliations:** From the ^1^Faculty of Life Sciences, University of Manchester, Manchester, UK; ^2^Paediatric Histopathology; ^3^Paediatric Surgery; ^4^Department of Paediatric Endocrinology, Royal Manchester Children’s Hospital, Central Manchester University Hospitals NHS Foundation Trust (CMFT), Manchester, UK.

**Keywords:** Islet, Congenital hyperinsulinism, Insulin, Hypoglycemia, Nucleomegaly, Serial block-face scanning electron microscopy, Pancreas, β cell, Hyperplasia

## Abstract

**Objectives:** To quantify islet cell nucleomegaly in controls and tissues obtained from patients with congenital hyperinsulinism in infancy (CHI) and to examine the association of nucleomegaly with proliferation.

**Methods:** High-content analysis of histologic sections and serial block-face scanning electron microscopy were used to quantify nucleomegaly.

**Results:** Enlarged islet cell nuclear areas were 4.3-fold larger than unaffected nuclei, and the mean nuclear volume increased to approximately threefold. Nucleomegaly was a normal feature of pediatric islets and detected in the normal regions of the pancreas from patients with focal CHI. The incidence of nucleomegaly was highest in diffuse CHI (CHI-D), with more than 45% of islets containing two or more affected cells. While in CHI-D nucleomegaly was negatively correlated with cell proliferation, in all other cases, there was a positive correlation.

**Conclusions:** Increased incidence of nucleomegaly is pathognomonic for CHI-D, but these cells are nonproliferative, suggesting a novel role in the pathobiology of this condition.

Congenital hyperinsulinism in infancy (CHI) is the most common cause of persistent or recurrent hypoglycemia in early childhood and infancy. CHI is broadly characterized by the inappropriate release of insulin from pancreatic β cells for the level of glycemia and is associated with hypoglycemia-induced brain injury and adverse long-term neurologic outcomes in more than one-third of cases.^1-3^ The hypoglycemia can be unresponsive to diazoxide, somatostatin analogues, and other medications, necessitating partial or near-total pancreatectomy.^1‐3^ Defects in several genes are identifiable causes of CHI,^4‐6^ but for many cohorts of patients with persistent disease, the genetic basis of disease is unknown,^7‐11^ ranging from 18% in Saudi Arabia^12^ to greater than 60% in Australia^13^ and China.^14^ The most common origins of drug-unresponsive disease are due to inactivating mutations in either the *ABCC8* or *KCNJ11* genes. These encode subunits of adenosine triphosphate (ATP)–sensitive K^+^ channels in β cells and result in loss of channel function, leading to inappropriate changes in the β-cell membrane potential, calcium influx, and insulin release.^15^

In addition to a spectrum of severities and genetic causes, CHI also has anatomopathologic diversity,^16^ which means that surgical management can be selectively deployed if affected parts of the pancreas can be identified. In patients with diffuse CHI (CHI-D), all islets throughout the pancreas are affected,^16^ whereas in patients with focal CHI (CHI-F), β-cell defects are localized to a topographical region caused by hyperplasia due to the loss of maternally imprinted genes.^17^ Recently, a third form of the condition has been described, accounting for approximately 10% to 15% of patients undergoing pancreatectomy: atypical CHI (CHI-A). Patients with CHI-A normally seek treatment later in the childhood period, have no known genetic cause of disease, and exhibit none of the histopathologic hallmarks of CHI-D or CHI-F.^18^ In CHI treatment centers with access to genotype screening facilities, genetic diagnosis of CHI can be helpful in distinguishing CHI-F from CHI-D prior to surgery because CHI-F is associated with a paternally inherited *ABCC8/KCNJ11* defect.^7^ Current techniques of imaging, including positron emission tomography–computed tomography (PET-CT) using 6-L-^18^F-fluorodihydroxyphenylalanine (^18^F-DOPA), can also be used to differentiate between CHI-F and CHI-D,^19^^,^^20^ but these are not widely available. For CHI-A, although measurements of serum incretin peptides may be of value,^21^ there is currently no preoperative investigation for the detection of this form of disease.

Intraoperative and postoperative diagnosis of CHI-F is based on the appearance of adenomatous hyperplasia of β cells within the focal lesion and is a clearly identifiable feature in cases of localized focal domains.^16^^,^^22^^,^^23^ In CHI-D, the islet architecture takes the form of ductal-insular complexes (nesidioblastosis) and has been reported to be associated with the appearance of nuclear enlargement in some islet cells.^24^^,^^25^ However, nesidioblastosis is a normal developmental feature of the early postnatal pancreas,^25^^,^^26^ and the detection of islet cell nucleomegaly is subjective and has not always been reported as pathognomonic of CHI-D.^24^^,^^27‐29^ With increased numbers of nontypical cases of CHI being encountered and reported in the literature,^18^^,^^21^^,^^23^^,^^30‐32^ we have investigated islet cell nucleomegaly in the postnatal pancreas and quantified the incidence of nucleomegaly in cases of CHI-F, CHI-D, and CHI-A. Our data have been generated using a combination of high-content analysis of postoperative tissues and serial block-face scanning electron microscopy to quantify nuclear volume changes in CHI and to identify the source of cells displaying islet cell nucleomegaly.

## Materials and Methods

### Human Tissue

Tissue samples were obtained from 17 patients with CHI. At the time of surgery, nine patients had CHI-D (aged 2-34 months), five had CHI-F (aged 2-10 months), and three had CHI-A (aged 12-36 months) Table 1. The diagnosis of CHI-F and CHI-D was made from established clinical, histopathologic, and ^18^F-DOPA PET-CT scan criteria^7^ and following the identification of mutations in either of the CHI-causing genes, *ABCC8* or *KCNJ11* (Table 1). Three patients had late-onset presentation of persistent CHI and received a PET-CT diagnosis of diffuse pancreatic involvement. However, all three patients were genotype negative for known defects in the CHI-causing genes *ABCC8*, *KCNJ11*, *HNF4A*, *HADH*, *GCK*, and *GLUD1.* Following 95% pancreatectomy, examination of the resected pancreas revealed a heterogeneous pattern of pancreatic histopathology consistent with CHI-A.^21^^,^^23^^,^^33^ Age-matched control tissues were obtained from eight individuals (aged 2 days, 7 weeks, 9 weeks, 4 months, 5 months, 6 months, 10 months, and 36 months) who died of nonpancreatic disease and showed unremarkable pancreatic histology.^26^ In all cases, pancreatectomy for CHI was performed at our center for alleviation of sustained hypoglycemia unresponsive to medical treatment. All pancreatic tissue for research was used in accordance with National Research Ethics Service (NRES) North West Committee approval, national codes of practice, and informed consent.

**Table 1:** Clinical Characteristics of the CHI Patient Cohort^a^

**Manuscript Code**	**Sex**	**Presentation**	**Age at Surgery, mo**	**Birth Weight, kg**	**Histology**	**Gene Defect**	**Genotype**
CHI-1	M	1 d	2	6.5	Diffuse	*ABCC8*	p.?(c.3992-9G>A)/p?(c.3992-9G>A)
CHI-2	M	1 d	2	4.1	Diffuse	*KCNJ11*	p.Q299R (c.896A>G)/p.Q299R (c.896A>G)
CHI-3	M	1 d	2	4.5	Diffuse	*ABCC8*	p.? (c.1818-?_1923+?del)/p.T172fs (c.512dup)
CHI-4	F	1 d	2	3.5	Diffuse	*ABCC8*	p.S581T (c.1741T>A)/p.?(c.3992-9G>A)
CHI-5	M	1 d	2	4.4	Diffuse	*ABCC8*	p.A30V(c.89C>T)
CHI-6	M	1 d	4	2.9	Diffuse	*ABCC8*	p.H36R (c.107A>G)/p.? (c.1630 + 1G>T)
CHI-7	M	1 d	6	2.9	Diffuse	*ABCC8*	p.? (c.148 + 1G>A)/p.? (c.148 + 1G>A)
CHI-8	M	1 d	6	1.9	Diffuse	*ABCC8*	p.I1512T (c.4535T>C)/? (AD)
CHI-9	F	1 d	13 and 34	4.6	Diffuse	*ABCC8*	p.? (c.4612-1G>T)/p.A4V (c.11C>T)
CHI-10	M	1 d	2	3.3	Focal	*ABCC8*	p.E128K, c.382G>A/None
CHI-11	M	1 d	3	3.9	Focal	*ABCC8*	c.3512delT/None
CHI-12	M	1 d	3	3.5	Focal	*ABCC8*	c.1879delC/None
CHI-13	F	5 mo	7	3.9	Focal	*ABCC8*	c.2116 + 1G>C/None
CHI-14	M	3 mo	10	3.6	Focal	*ABCC8*	c.2995C>T/None
CHI-15	M	7 mo	12	3.4	Atypical	Unknown	Unknown
CHI-16	F	11 mo	17	2.6	Atypical	Unknown	Unknown
CHI-17	M	30 mo	36	3.6	Atypical	Unknown	Unknown

CHI, congenital hyperinsulinism in infancy; p.?, intronic mutation resulting in unknown protein.

^a^All patients were treated for hypoglycemia and classified as having diffuse, focal, or atypical CHI based on clinical characteristics, including the age of presentation of symptoms (“Presentation”), genotyping, positron emission tomography–computed tomography diagnosis, or pancreatic histology following surgery. All patients underwent surgery to alleviate hyperinsulinism.

### Immunohistochemistry and Nuclear Analysis

Immunohistochemistry was performed as described previously on 5-µm-thick sections of tissue.^26^ All tissues were fixed in 4% paraformaldehyde within 5 minutes of retrieval and embedded in paraffin wax. For high-content assessment of nuclear size, each section was digitized by a ×20/0.80 Plan Apo objective using the 3D Histech Pannoramic 250 Flash II slide scanner (3DHISTECH, Budapest, Hungary). Pannoramic Viewer and HistoQuant software packages were then used to quantify nuclear areas (3DHISTECH).^26^ For each tissue sample, we selected a minimum of 20 islets with clear boundaries and quantified the number of visible nucleomegalic cells as a fraction of the total cell count within the designated region. Islets from nonlesion domains were not located within the margins of the focal lesions. Enlarged nuclei from the endocrine regions, which were identified as three times larger than the surrounding nuclei, were randomly selected, and the areas were calculated through the software using edge detection. Normal-sized nuclei from both endocrine and exocrine regions were measured in the same manner. This method of quantification was preferred to that of measuring the nuclear radius^24^ since nuclei are not uniform, particularly those exhibiting nucleomegaly. For CHI-F tissue, the nuclear dimensions of endocrine cells within lesions were quantified from regions of interest involving a minimum of 500 cells. In addition to the analysis of enlarged nuclei on the surface of tissue sections, we also examined expression in entire islet structures. For this, fifty 5-μm serial sections of tissue were cut from CHI-D and control tissue blocks, which were then scanned, and the images were used to digitally reconstruct the tissue block for quantitative analysis (HistoQuant; 3DHISTECH). This was used to determine the total numbers of cells with enlarged nuclei within the entire islet structure. Cells undergoing proliferation were identified using Ki-67 immunohistochemically stained slides (monoclonal, 1:100; Novocastra, Milton Keynes, UK); apoptosis was investigated using cleaved caspase 3 fluorescence (polycolonal, 1:50; Cell Signaling, Leiden, Switzerland).

### Transmission Electron Microscopy and Serial Block-Face Scanning Electron Microscopy

Tissue samples were fixed and processed using a high-density staining method suitable for block-face imaging as previously described.^34^ Briefly, samples were fixed in 4% formaldehyde (Sigma, Gillingham, UK) and 2.5% glutaraldehyde (Agar Scientific, Stansted, UK) in 0.1 M HEPES buffer (Fisher Scientific, Loughborough, UK) (pH 7.2) overnight. They were then postfixed with 1% osmium tetroxide (Agar Scientific) and 1.5% potassium ferrocyanide (BDH Chemicals, Milton Keynes, UK) in 0.1 mol/L cacodylate buffer (Fisher Scientific) (pH 7.2) for 1 hour, followed by 20 minutes in 1% thyocarbohydrazide (Sigma) solution and 30 minutes in 1% osmium tetroxide. After that, samples were incubated in 1% uranyl acetate (Fisher Scientific) at 4°C overnight. The next day, samples were stained with Walton^35^ lead aspartate for 30 minutes at 60°C and then dehydrated in ethanol series. Subsequently, samples were infiltrated with TAAB 812 hard-grade resin (TAAB Laboratories, Reading, UK) and polymerized for 24 hours at 60°C. After the preparations, ultrathin sections (70 nm) were cut with a Reichert Ultracut ultramicrotome (Reichert Ultracut, Vienna, Austria) and observed with a FEI Tecnai 12 Biotwin microscope (FEI, Hillsboro, OR) at 100 kV accelerating voltage. Images were taken with a Gatan Orius SC1000 CCD camera (Gatan, Abingdon, UK) and analyzed with Fiji software (http://fiji.sc).^36^

For serial block scanning,^37^ samples were trimmed and mounted onto an aluminum cryo specimen pin using superglue (cyanoacrylate; Permabond, Winchester, UK). Care was taken to orient the sample so that the imaging plane was perpendicular to the pin axis. The sample was then trimmed to form a trapezoid face approximately 500 × 500 × 150 µm. The face of the trapezoid was polished on an ultra-microtome before mounting on a Gatan3View (Gatan) within an FEI Quanta 250 FEG scanning electron microscope. The machine allows serial section transmission electron microscopy (TEM)–like images to be collected in an automated fashion. For the purpose of this study, the imaging settings were as follows: accelerating voltage, 3.8 kV; spot size, 3.5; final lens aperture, 30 µm; chamber pressure, 66 Pa; and quadrant magnification, ×3,500. This gave a horizontal field width of approximately 40 µm, with image dimensions 4,096 × 4,096, a pixel dwell time of 10 µs, and cut thickness of 100 nm. Individual image intensities were floated to a common mean and SD to remove variation in beam intensity or detector sensitivity that can occur during long data acquisitions.^38^ Some imaging noise was removed by a standard two-dimensional Gaussian smoothing using a 3 × 3 kernel to aid manual segmentation. Raw data files were converted to an MRC file stack using IMOD Software (Boulder, CO; http://bio3d.colorado.edu/imod/), which was also used in three-dimensional reconstruction of nuclei.^38^^,^^39^

### Statistical Analysis

Data are presented as mean ± standard error, and a one-way analysis of variance was used to determine whether there are any significant differences between the means of data sets.

## Results

### Quantification of Islet Cell Nucleomegaly

**Image 1:**
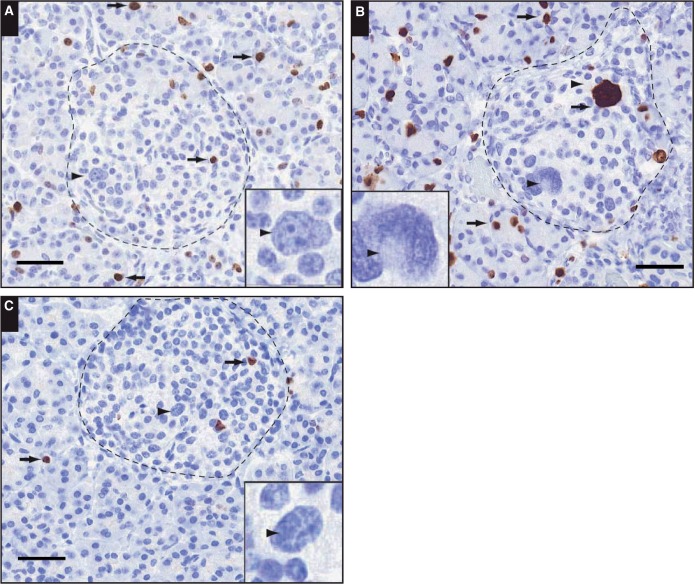
Typical images of nucleomegaly (arrowheads and insets) in islets (dotted lines) from tissue sections obtained from different patients with congenital hyperinsulinism in infancy (CHI): CHI-6 (**A**), CHI-4 (**B**), and a 10-month-old control, (**C**). Tissue sections were stained using the proliferation marker Ki-67, and typical Ki-67–positive cells are illustrated by the arrows. Note that not all Ki-67–positive nuclei are enlarged and that not all enlarged nuclei are positive for Ki-67. Scale bar is equivalent to 40 μm (insets, ×2.5).

**Image 2:**
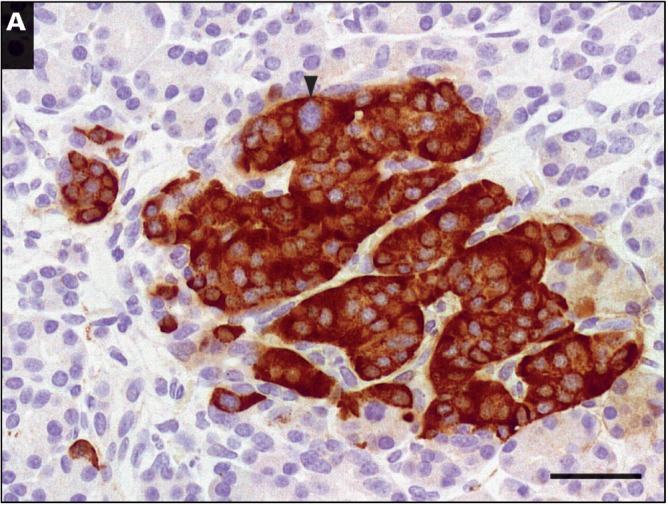

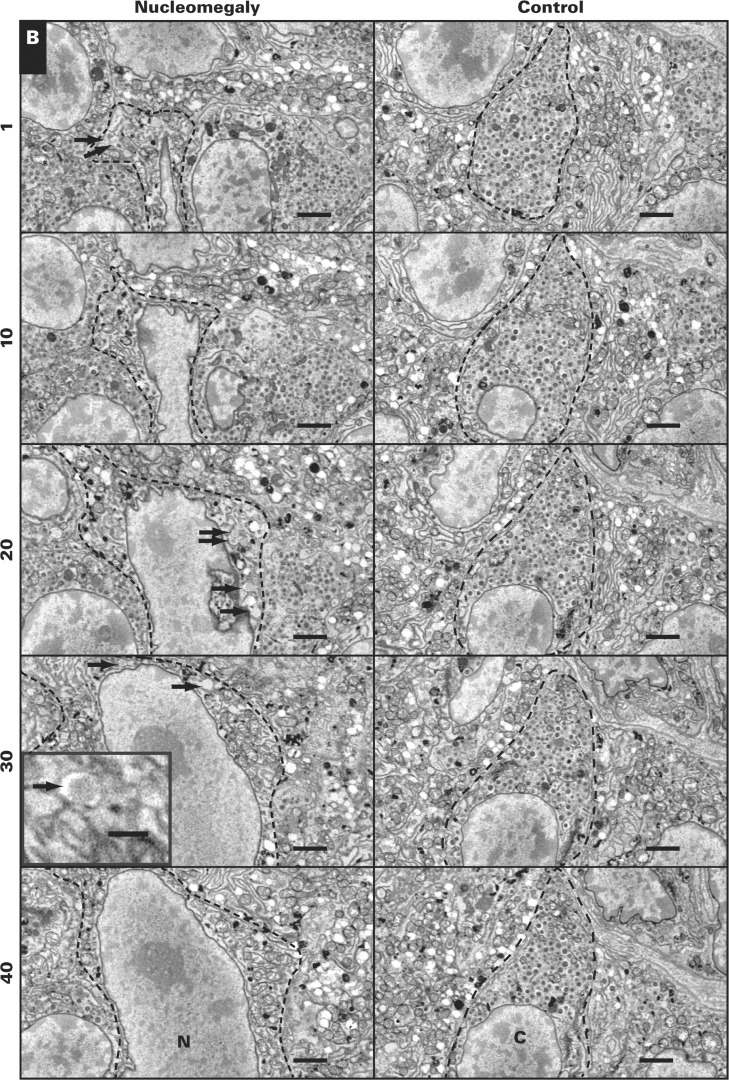
Islet cells with nucleomegaly have an endocrine phenotype. **A**, Staining in a congenital hyperinsulinism in infancy (CHI) islet with nucleomegaly indicated by the arrowhead. Scale bar is equivalent to 40 μm. **B**, A series of images obtained by serial block-face scanning electron microscopy through adjacent parts of the tissue. Note the limited number of secretory granules (indicated by arrows) in the cell with nucleomegaly (N) compared with the surrounding cells and the control cell illustrated in the right-hand montage (C). Each image is separated by 1 μm. Scale bars are equivalent to 2 μm; expanded image 500 nm. The data were obtained from patients CHI-9 (**A**) and CHI-11 (**B**).

**Figure 1:**
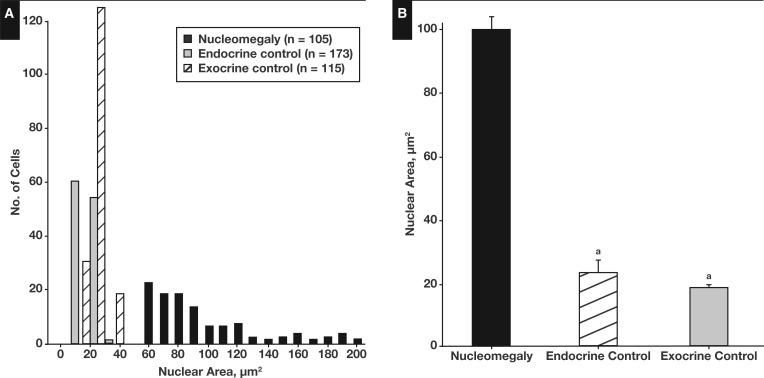
Estimates of the surface area of nuclei in congenital hyperinsulinism in infancy and control cells. **A**, The ranges of nuclear areas from 393 cells; exocrine cells had a small range of nuclear areas compared with endocrine cells, and both were much smaller than the distribution of nuclear areas from islet cells exhibiting nucleomegaly. **B**, Enlarged nuclei in islet cells had an area of 101.1 ± 3.8 µm^2^ (mean ± SEM, n = 105) compared with 23.0 ± 0.4 μm^2^ (n = 173) and 18.7 ± 0.3 μm^2^ (n = 115) in control endocrine and exocrine cells, respectively. ^a^*P* < .001.

**Figure 2:**
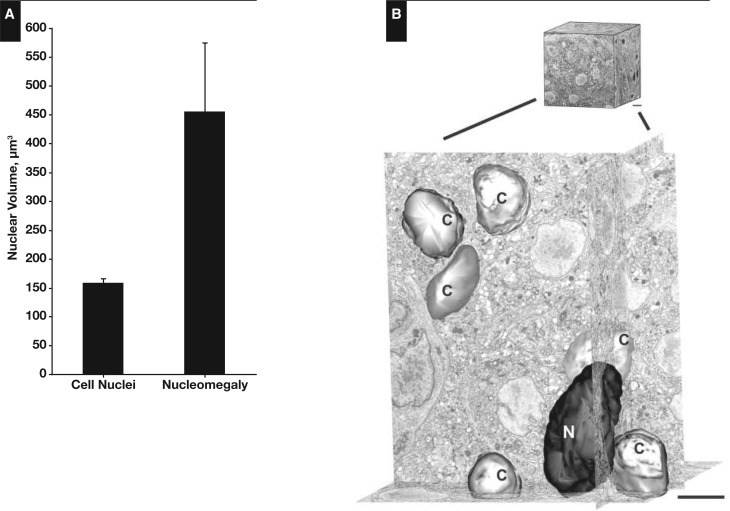
Nuclear volume estimates in congenital hyperinsulinism in infancy (CHI) islet cells. **A**, Summary of the volume estimates of control nuclei (mean ± SEM; 162.32 ± 8 μm^3^, n = 30) and enlarged nuclei (452.83 ± 119.8 μm^3^, n = 4) in islet cells. The data were obtained from three patient samples with CHI. **B**, Transmission electron microscopy data and a digital reconstruction of control islet cell nuclei (C) and a cell exhibiting nucleomegaly (N), using serial block-face scanning electron microscopy. A total of 485 serial sections of tissue in 100-nm thicknesses were used to generate the data set. Scale bar is equivalent to 2 μm. Data obtained from patient CHI-11.

Islet cell nucleomegaly has a distinct cytomorphic appearance Image 1 and is typified by an increase in the area of the nucleus compared with the nuclei of cells in the surrounding area.^25^ Here, we found that the mean area of enlarged nuclei in islet cells (100.1 ± 3.8 μm^2^, n = 105) was 4.3-fold larger than nuclei in control endocrine cells (n = 173) and 5.3-fold larger than nuclei in exocrine cells (n = 115) Figure 1. Figure 1A summarizes the range of nuclear areas associated with enlargement, which can be 10-fold greater than control cells, leading to the appearance of giant nuclei (Image 1B). To quantify the nuclear volume of islet cells, we digitally reconstructed the nuclei of cells from TEM images of the tissue block following 100-nm serial sectioning. From this, the mean volume of nuclei of islet cells was estimated to be 162.08 ± 7 μm^3^ (n = 30). This value is entirely consistent with data obtained from rodent islets using the same procedure.^40^ Islet cells with nucleomegaly are rare (see below), but image stacks were obtained from which cells with enlarged nuclei were observed, and the mean nuclear volume was estimated to be 453.83 ± 119 μm^3^ (n = 4) (Figure 2 and supplementary data; all supplementary materials can be found at *American Journal of Clinical Pathology* online). Several other putative enlarged cells were identified but excluded from the analysis on the basis that they did not contain a fully resolved structure within the complete image stack. Islet cells with enlarged nuclei appear to have an endocrine phenotype since they stained positive for the neuroendocrine cell marker chromogranin (n = 398/405 cells) Image 2A. However, the cells displaying nucleomegaly contained a limited number of secretory granules compared with control endocrine cells Image 2B.

### Occurrence of Islet Cell Nucleomegaly

**Figure 3:**
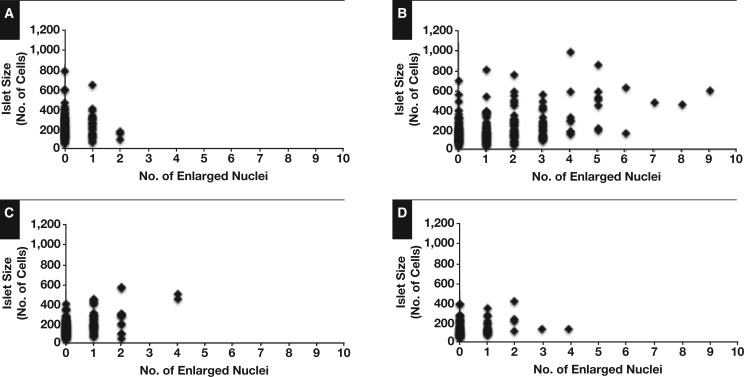
The incidence of nucleomegaly in islet cells. These panels summarize the range of enlarged nuclei observed in islet structures using tissue sections for each congenital hyperinsulinism in infancy (CHI) cohort and age-matched control tissue. The number of enlarged nuclei per islet surface area has been expressed relative to the number of cells within the islet. Only diffuse CHI (CHI-D) islets (**B**) had a high incidence of more than four enlarged nuclei per islet surface area. Indeed, some CHI-D islets were found to contain up to nine nucleomegalic cells in a single field of view. **A**, Control. **B**, CHI-D. **C**, Atypical CHI. **D**, Focal, control.

High-content analysis was performed to quantify the occurrence of islet cell nucleomegaly. These data have then been used to describe the incidence of nucleomegaly both within islets and as a fraction of the total endocrine cell population. Since CHI-F is not composed of islet structures, regions of interest of more than 500 cells were randomly selected within the focal lesion for analysis. Table 2 summarizes our findings. In neonatal control tissue, we found that 0.1% ± 0.01% of islet cells (n = 26,847) had enlarged nuclei and that these could be detected in 22% of islets (n = 117). However, only 2% of the islets had two or more cells with enlarged nuclei, and there were no islets with three or more cells with nucleomegaly (Table 2). In marked contrast, the occurrence of islet cell nucleomegaly was significantly higher in islet cells from patients with CHI-D (0.7% ± 0.1%, n = 40,320; *P* < .001), and enlarged nuclei could be detected in islet structures far more readily. Thus, we found that approximately 70% of CHI-D islets had one or more enlarged cell nuclei and that almost 25% of CHI-D islets were associated with three or more nucleomegalic cells. While nucleomegaly could be detected in all forms of CHI, overall there were no significant differences in incidences between control, CHI-A, and CHI-F. Interestingly, in the lesion domains of CHI-F tissue, the incidence of nucleomegaly was eightfold lower than in CHI-D despite the fact that the genetic cause of uncontrolled insulin release is the same. Figure 3 summarizes the ranges of enlarged nuclei per islet in controls and the CHI patient groups. The data show that the appearance of multiple nucleomegaly per islet and specifically more than five enlarged nuclei is a defining feature of CHI-D. We found no relationship between islet cell nucleomegaly and the number of cells per islet (Figure 3; *r*^2^ = –0.001, n = 395).

**Table 2:** Occurrence of Islet Cell Nucleomegaly in CHI

Characteristic	**Control**	**Diffuse CHI**	**Focal CHI**		**Atypical CHI**
			**Lesion**	**Nonlesion Domain**	
No. of cases	6	9	4	5	3
No. of islets	117	179	NA	80	145
No. of cells	26,847	40,320	47,598	12,576	24,590
Islet cells with enlarged nuclei, %	0.10 ± 0.01	0.67 ± 0.1^b^	0.08 ± 0.02	0.28 ± 0.06	0.22 ± 0.09
Islets with ≥1 enlarged nuclei, %	22 ± 0.4	71 ± 6^c^	NA	32 ± 5	32 ± 14.7
Islets with ≥2 enlarged nuclei, %	2 ± 1.6	45 ± 7^c^	NA	8 ± 2	8 ± 4.3
Islets with ≥3 enlarged nuclei, %	0	24 ± 6^c^	NA	3 ± 1.4	1 ± 1.1

CHI, congenital hyperinsulinism in infancy; NA, not applicable.

^a^Summary data for the occurrence of enlarged nuclei in both islet structures. (n = 80-179) and in the population of islet cells (n = 12,576-47,598) from either control or CHI tissues. For cases of focal CHI, data were obtained from the lesion and the nonlesion domains of the pancreas. Values are presented as mean ± SEM unless otherwise indicated.

^b^*P* < .001.

^c^*P* < .0001 vs control values.

**Image 3:**
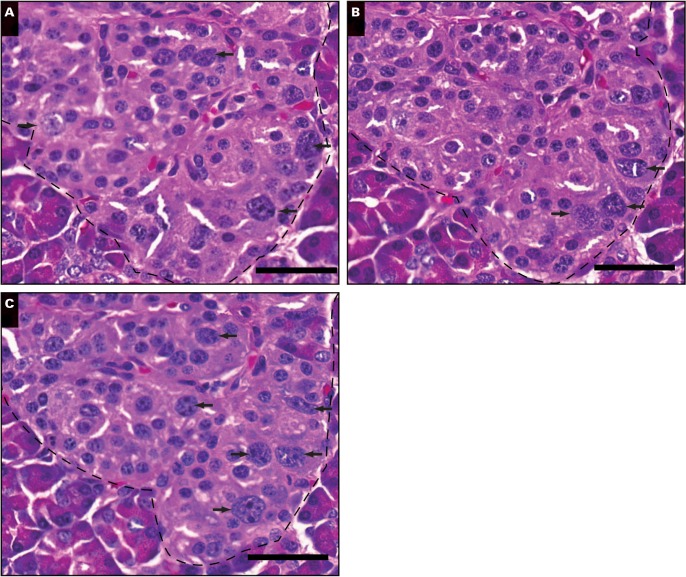
**A-C**, Serial sections of tissue to illustrate the presence of multiple enlarged nuclei, indicated by arrows. Note that most of the nuclei appear in successive 5-μm sections of tissue. Data were obtained from patient CHI-9. Scale bar is equivalent to 30 μm.

**Figure 4:**
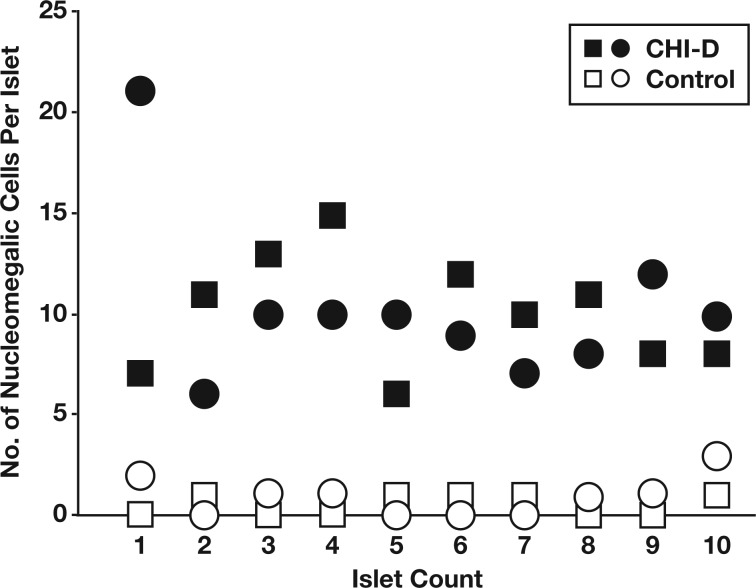
Multiple islet cell nucleomegaly in diffuse congenital hyperinsulinism in infancy (CHI-D) islets. An analysis of multiple enlarged nuclei in control (open shapes) and CHI-D islets (closed shapes). The comparative data set was obtained from a patient with congenital hyperinsulinism in infancy at two time points following two pancreatectomies—one at 13 months (squares) and the other at 30 months of age (circles). Fifty serial sections of tissue were used to assess total islet cell nucleomegaly from a minimum of 10 islets. Note how 100% of CHI-D islets had enlarged nuclei (n = 20). The minimum number of cells with nucleomegaly was six per islet, and one islet was found to contain 21 nucleomegalic cells. By comparison, fewer control islets contained nucleomegalic cells.

The appearance of multiple enlarged nuclei per islet was confirmed by serially sectioning pancreata from control and CHI-D tissues and quantifying the total number of nucleomegalic cells per islet rather than detecting the surface expression of enlarged nuclei in tissue sections. The CHI-D data in Figure 4 and Image 3 come from the same patient who underwent two surgeries at 13 months and 34 months of age. The data show that all CHI-D islets (n = 20) were positive for enlarged nuclei compared with 55% of control islets (n = 11/20). On average, we found 10.2 ± 0.8 islet cells with enlarged nuclei in CHI-D islets (n = 20) compared with 0.7 ± 0.2 cells per islet in controls (n = 20). Since we found no differences in the incidence of nucleomegalic cells in the CHI tissues following both procedures, these data imply that islet cell nuclear enlargement is not a developmental feature of the pancreas but a defining hallmark of islet pathobiology.

### Islet Cell Nucleomegaly and Proliferation

Since nuclear enlargement might be as a consequence of chromatin decondensation in preparation for cell division, we next examined the correlation of Ki-67 staining (as a marker of proliferation) with nucleomegaly. In age-matched control islet cells (n = 16/30) but not adult islets (0%, n = 0/18), 53% of cells with nucleomegaly were proliferating. Surprisingly, in cases of CHI-D, enlarged nuclei appear to be negatively correlated with Ki-67 expression as only 9% of cells with nucleomegaly were Ki-67 positive (n = 27/291; see Image 1). By comparison, 67% of CHI-F (n = 22/33), 44% of CHI-A (n = 23/57), and 67% (n = 20/30) of nucleomegalic endocrine cells in nonlesion domains of focal CHI cases were also found to be Ki-67 positive. Finally, nucleomegaly was not associated with apoptosis since enlarged nuclei were negative for cleaved caspase 3, a marker for the detection of apoptosis (n = 15).

## Discussion

CHI is a diverse disease involving different genetic causes affecting proteins such as glutamate dehydrogenase, hepatic nuclear factors 4α and 1α, the inwardly rectifying potassium channel Kir6.2, monocarboxylic acid transporter 1, short-chain 3-OH acyl-CoA dehydrogenase, the sulfonylurea receptor 1, and uncoupling protein 2. The disease ranges in severity and duration of hypoglycemia and histopathologic origins. Over the past decade, advances in genetic and imaging procedures have contributed to major changes in the short- and long-term management of patients with CHI. This has been most striking for patients with CHI-F. Detection of paternally inherited mutations in K_ATP_ channel genes and the use of imaging techniques have now raised the possibility for many patients that a subtotal pancreatectomy/lesionectomy can be used to excise the lesion and cure the disease.^4^^,^^6^^,^^41‐44^ For patients with CHI-D and CHI-A unresponsive to medical therapy, surgical options are limited to a near-total or partial pancreatectomy, respectively.^43^^,^^44^ While this can alleviate the problems of hypoglycemia in the short term, extensive surgery will also predispose patients to iatrogenic diabetes. Intraoperative diagnosis of the extent of pancreatic pathology is now routinely required to confirm initial investigations, and this can be challenging for the pathologist if the focal lesion has poorly defined margins, the patient has CHI-A,^18^^,^^21^^,^^23^^,^^30‐32^^,^^42^ or the child is very young when the normal developmental features of the pancreas are altered in CHI^26^ and may mask pathologic hallmarks.

For more than 30 years, the presence of enlarged nuclei in islet cells has been pathognomonic for CHI,^23^ although the frequency and extent of nucleomegaly in the different forms of CHI have not been investigated. In our study, nucleomegaly was detected not only in each of the different forms of CHI but also in age-matched control islets and the healthy regions of the pancreas of children with CHI-F. While the overall proportion of cells with nucleomegaly was small (0.1% of the total endocrine cell population), these cells were readily detectable in 22% of control islets. The incidence of islet cell nucleomegaly was always higher in tissue from patients with CHI than from controls, with similar profiles of expression being found in islets from patients with CHI-A and CHI-F. This implies that islet cell nucleomegaly should be used cautiously to diagnose CHI-A or unusual cases of CHI as part of an intraoperative or routine histologic procedure. Our analysis strongly reaffirms the role of islet cell nucleomegaly as a hallmark of CHI-D when enlarged nuclei are detected in more than one-third of islets (we found 71% of CHI-D islets were affected) and when more than one enlarged cell nucleus is detected in no fewer than 10% of islets. In practice, we found that CHI-D islets were almost six times more likely to have more than one nucleomegalic cell and that CHI-D would often be associated with many more. While several groups have reported that nucleomegaly is not consistently seen in CHI-D cases,^24^^,^^27^^,^^29^ our observations do not support this view. In our study, not only did we detect nucleomegaly in all cases of CHI-D, but we also found similar profiles of expression in each of the cases. We also found that the rate of detection was unaltered when the same patient underwent a second procedure more than 20 months after the first pancreatectomy. This has not been previously reported and indicates that there is a close relationship between nucleomegaly and the pathobiology of islet cells in CHI, particularly CHI-D. It seems unlikely that islet cell nuclear enlargement is caused by *ABCC8* gene defects since the incidence of nucleomegaly is eightfold lower in focal compared with diffuse disease. Similarly, we found no difference in the incidence of nucleomegaly between *ABCC8* and *KCNJ11* as a genetic cause of CHI.

In a recent publication, we explored the type of islet cells associated with nucleomegaly in CHI. We described that these cells were often positive for the expression of insulin but not always and sometimes stained positive for somatostatin expression but not for glucagon, pancreatic polypeptide, or ghrelin.^26^ The endocrine lineage of the nucleomegalic cells is confirmed here by demonstrating that they are consistently associated with the expression of the neuroendocrine marker chromogranin, but in addition, we found that these cells have significantly fewer secretory granules than controls. This was demonstrated by serial block-face scanning electron microscopy, which allowed us to image the entire ultrastructure of the cell rather than just a single field of view. The reason why these cells possess so few secretory granules is not clear, but it may reflect an immature phenotype and explain why some groups report that insulin cannot be detected in cells with nucleomegaly.

The presence of enlarged nuclei in CHI cells has been implicated in cell proliferation, and since enhanced proliferation of the endocrine pancreas is a feature of focal and diffuse CHI,^22^^,26,29,45^ we have closely examined the association of Ki-67 staining. In neonatal control tissues, approximately 50% of cells with an enlarged nucleus also stained positive for Ki-67 expression. This suggests a positive association with expansion of the islet cell mass in the first 12 months following birth and is supported by our observations that while nucleomegaly can be occasionally detected in adult islets, it is never associated with Ki-67 expression. In CHI-F endocrine cells, presumably because of the loss of the cell cycle repressor p57kip2,^17^ 67% of cells were also positive for Ki-67 expression, but surprisingly, less than 10% of the nucleomegalic cells in islets from CHI-D tissue were positive for Ki-67 expression. While these data are consistent with our recent findings in CHI-D that not all nucleomegalic cells were positive for the cell cycle enhancers CDK6 and pRb,^26^ the finding that 90% of the cells are not expressing a marker of proliferation is intriguing. In contrast to CHI-D islets, more than 65% of islets from the region of the pancreas outside the focal lesion have enlarged nuclei that are positive for Ki-67 expression. Since these islets are widely considered to be inactive or “resting” in CHI-F,^24^^,^^30^^,^^46^ our data suggest that this is not the case, and their role in the pathobiology of CHI warrants further investigation. We also found that 44% of nucleomegaly cells in CHI-A were undergoing proliferation. The pathobiology of CHI-A is undetermined at this stage, but these data suggest that at least part of the disease profile is associated with increases in cell turnover.

In summary, we support the role of islet cell nucleomegaly as a diagnostic hallmark of CHI-D, although the role of these cells in the disease process is still undetermined. Our data indicate that the detection of more than two enlarged nuclei in as few as 10% of islets is pathognomonic for CHI-D. Since islet cell nuclear enlargement is a normal feature of the control pancreas and is detected in the nonlesion domains of CHI-F tissue, this needs to be taken into consideration when nucleomegaly is used as a diagnostic marker during intraoperative diagnoses of other forms of CHI, including focal and atypical disease.

## Supplementary Material

supplementary data
