# Supplementary material for: Enhanced Islet Cell Nucleomegaly Defines Diffuse Congenital Hyperinsulinism in Infancy but Not Other Forms of the Disease
Source: Am J Clin Pathol. 2016 Jun 22;145(6):757–68. doi: 10.1093/ajcp/aqw075 (PMC4922485; doi:10.1093/ajcp/aqw075)
Supplement: supplementary data [file ajcp_aqw075_index.html]

Supplementary Data | American Journal of Clinical Pathology

## Supplementary Data

files

- Supplementary Data - avi file
